# Photo-Attachment of Biomolecules for Miniaturization on Wicking Si-Nanowire Platform

**DOI:** 10.1371/journal.pone.0116539

**Published:** 2015-02-17

**Authors:** He Cheng, Han Zheng, Jia Xin Wu, Wei Xu, Lihan Zhou, Kam Chew Leong, Eugene Fitzgerald, Raj Rajagopalan, Heng Phon Too, Wee Kiong Choi

**Affiliations:** 1 Advanced Materials for Micro- and Nano- Systems, Singapore-MIT Alliance, Singapore, Singapore; 2 Department of Electrical and Computer Engineering, National University of Singapore, Singapore, Singapore; 3 NUS Graduate School for Integrative Sciences and Engineering, National University of Singapore, Singapore, Singapore; 4 Bioprocessing Technology Institute, 20 Biopolis Way, #06-01 Centros, Singapore, Singapore; 5 GLOBALFOUNDRIES Singapore Private Limited, Singapore, Singapore; 6 Department of Materials Science and Engineering, Massachusetts Institute of Technology, Cambridge, Massachusetts, United States of America; 7 Skolkovo Institute of Science and Technology, Moscow, The Russian Federation; 8 Department of Biochemistry, National University of Singapore, Singapore, Singapore; Osaka University, JAPAN

## Abstract

We demonstrated the surface functionalization of a highly three-dimensional, superhydrophilic wicking substrate using light to immobilize functional biomolecules for sensor or microarray applications. We showed here that the three-dimensional substrate was compatible with photo-attachment and the performance of functionalization was greatly improved due to both increased surface capacity and reduced substrate reflectivity. In addition, photo-attachment circumvents the problems induced by wicking effect that was typically encountered on superhydrophilic three-dimensional substrates, thus reducing the difficulty of producing miniaturized sites on such substrate. We have investigated various aspects of photo-attachment process on the nanowire substrate, including the role of different buffers, the effect of wavelength as well as how changing probe structure may affect the functionalization process. We demonstrated that substrate fabrication and functionalization can be achieved with processes compatible with microelectronics processes, hence reducing the cost of array fabrication. Such functionalization method coupled with the high capacity surface makes the substrate an ideal candidate for sensor or microarray for sensitive detection of target analytes.

## Introduction

Biosensors and microarray has been a valuable tool for biomedical research, and most devices are based on solid phase supporting substrate where biomolecules are immobilized at predefined positions for location registered reading. A major challenge of this technology is to produce reliable and inexpensive substrates that give high signal-to-noise (SNR) detection of analytes. We have recently discovered a unique silicon (Si) nanowire based platform [1], fabricated by the glancing angle deposition-metal assisted chemical etching (GLAD-MACE) technique that can accommodate an extremely large number of sense-DNA for the addressing of captured analytes. We have attributed the 3-D and unique porous nature of the GLAD-MACE nanowires for the enhanced loading capacity of the platform [1,2].

With conventional substrates, hydrophobically functionalized surfaces have been commonly used for fabrication. With a hydrophobic surface, it is easier to produce smaller spots [3] which will be advantageous in two ways: first, a simple reduction in spot size may increase signal intensity when detecting target at low concentration [4–6], and secondly, for device require high throughput, a large number of sites can be housed on a single substrate. In addition, as the droplet spreading is limited, uneven crystallization of buffer salt due to evaporation is also minimized which gives better spot uniformity. Furthermore, hydrophobic surface will suppress strong convection at the edge of the droplet, avoiding forming doughnut shaped spots, again improving spot uniformity [7]. On the other hand, substrate with 3D structures that is hydrophilic will allow a complete wetting of aqueous solution to the substrate surface and hence maximize biomolecules attachment or capturing to the substrate. For examples, wicking has been reported on micro structured surfaces [8], porous surfaces [9] and fibrous surfaces [10]. The main problem is that such substrates wick significantly and resulted in difficulty in controlling spot size and geometry. To miniaturize sites on hydrophilic surfaces, organic solvent, such as DMSO, has been used to alter the wetting characteristics of liquid [11]. However, this may result in denature of biomolecules. Another method is to cover the substrate surface with photoresist and selectively removed the photoresist at locations for subsequent printing [12]. However, a complete removal of photoresist and possible leaching of photoresist can be potential problems for this method. The GLAD-MACE platform is highly hydrophilic so while it can fully reap the benefits of increased surface area (due to 3D and porous surface) for attachment of analytes, extending the detection limit to fmol region without amplification, wicking is a severe problem for the miniaturization of the GLAD-MACE platform. The same is true generally for any hydrophilic substrate with 3D micro- or nanostructures as researchers continue to find innovative ways to combat the problem [13]. In this study, we aim to show the size detection sites can be shrink on superhydrophilic wicking surfaces with the example of GLAD-MACE substrate.

There are reports for the photo-attachment of biomolecules on hydrophobic surfaces. For examples, photo cross-linking of DNA and protein molecules on gold [14], TiO_2_ [15], GaN [16], quartz [17], diamond [18] and polymers [19] has been attempted as potential problems such as moisture content, printing buffer composition, missing immobilization spot or tailing of immobilization spot, that are often associated with microarray printing, can be avoided using this technique [20]. In this paper, we first examine if photo-attachment of biomolecules can be applied to the GLAD-MACE platform and compare the efficiency of photo-attachment process to the conventional chemical activation process. We then present results of a comprehensive study on the influences of the various factors (e.g. chemicals, adhesion, hybridization etc.) on photo-attachment of analytes on the GLAD-MACE platform. Note that in this study, we have purposely use simple lithography setup and commercially available chemicals of bifunctional psoralen and diazirine, which can intercalate with nucleobase or form C-H or N-H bond insertions [21], so that we can achieve the miniaturization of the detection spots with standard Si processing technology and commonly available chemicals to demonstrate that GLAD-MACE devices can be produced cost effectively in large quantity, and photo-attachment is a feasible approach to circumvent wicking while working with hydrophilic 3D substrates.

## Experimental

The fabrication of GLAD-MACE Si nanowires has been reported in our previous study [1,22]. Briefly, Si wafers were subjected to the GLAD deposition of gold. Due to the shadowing effect, gold nanoparticles were formed on the surface of Si wafers. The deposited wafers were then etched in a mixed solution of HF and H_2_O_2_, where Si under gold particles would be selectively etched away via the MACE mechanism [23,24]. After etching, the remaining gold was removed by gold etchant, and the substrates were subsequently dried with nitrogen gas and oxidized in oxygen at 900°C for 35 minutes an oxidation furnace.

The oxidized silicon surface was aminated with 2% (3-Aminopropyl)triethoxysilane (APTES) in absolute ethanol at room temperature. The substrates were submerged in the solution and incubated on an orbital shaker for 12 hours. The substrates were then washed with absolute ethanol for 3 times, 10 minutes each to removed excessive APTES. Finally, the washed substrates were placed in a 70°C oven for 24 hours before use. The amount of free amine on the nanowire surface was estimated with fluorometry to provide information on the amount of reagent needed for subsequent experiments. The aminated substrates were reacted with NHS-Dylight 550 for 4 hours at 1 μM at room temperature. The substrates were then washed with 0.1% SDS to 60°C for 30 minutes vigorously, after which the washing solution was collected and its florescence measured. The excitation and emission spectrum of Dylight 550 was calibrated with 0.1% SDS in HPLC water at concentrations below 1 μM.

N-hydroxysuccinimide (NHS)-Psoralen was dissolved in DMSO at a concentration of 4.5 mg/ml and kept at -20°C. NHS-Diazirine was dissolved in phosphate buffered saline (PBS) at pH 7.5 at a concentration of 2.33 mg/ml and kept at -80°C. The aminated substrates were cleaned and the area of nanowires was estimated. 160 nmol/cm^2^ of psoralen or diazirine was used for functionalization. NHS-Psoralen was further dissolved in 50mM HEPES buffer at pH 8.2 before reaction. Crosslinking of psoralen and diazirine to the nanowire surface was performed at room temperature in dark environment for 8 hours. After crosslinking, diazirine functionalized substrates were washed with PBS and ethanol; and psoralen functionalized substrates were rinsed with DMSO and ethanol, both types of substrates were air dried before storage.

Surface passivation was performed by crosslinking adipic acid to functionalized substrates. Adipic acid was dissolved in 0.1 M MES buffer to a weight percentage of 1.3%. The pH of the solution was adjusted to 6 with NaOH. Subsequently, adipic acid solution was reacted with nanowire surfaces in the presence of 0.25 M 1-Ethyl-3-(3-dimethylaminopropyl)carbodiimide (EDC) in MES buffer. Reactions were proceeded for 2 hours, and terminated by washing with 1M Tris buffer for 10 minutes. The substrates were subsequently washed with PBS and dried with absolute ethanol.

Photo-attachment was carried out with a UV lamp with an output power of 8W at 254 nm, 302 nm and 365 nm (UVP, parts No. 34-0007-01, 34-0042-01 and 34-0006-01 respectively). Irradiation was carried out 2.5 cm away from substrate surface. 100 ml of activation buffer was constituted with 57 μL of 100% acetic acid and 0.136 g sodium acetate at pH adjusted to 3.6. The dye labelled oligo was dissolved ether in PBS or in activation buffer with 2% Triton X-100 (TX100) for photo crosslinking reaction. The functionalized substrates were either subjected to UV treatment or placed in dark as reference. The set up was kept in a humid environment to avoid evaporation of liquid. Various time points were taken during photo-attachment to measure the time response of crosslinking. For experiment other than crosslinking time course study, the exposure time was set to 40 minutes. From our crosslinking time course experiments (see S1 File), we found that diazirine may have higher reactivity for photo-attachment than psoralen.

For chemical crosslinking, 0.25M EDC was used to conjugate aminated sense oligos (20 μM) to carboxylated GLAD-MACE substrates in 0.1M MES buffer at pH 6. The substrates were placed in a humid chamber for 2 hours at room temperature. For comparison, the same oligonucleotide strand with the same concentration was used in activation buffer.

For chemical crosslinking and photo-attachment, washing was done with 0.1% SDS at 60°C for 10 minutes, and followed by a 10 minutes wash with 1M Tris buffer at pH 8.5. The substrates were finally dried with ethanol.

Hybridization was performed in PBS with 2% TX100 at room temperature at various target concentrations for 1 hour on a stationary stage. At the end of incubation, excessive reagent was decant and substrates were washed with PBS with 2% TX100 for 10 minutes, and followed by PBS for 10 minutes each. The substrates were finally washed with absolute ethanol and then air dried. The sequences for the probe and targets are as follow (5’ to 3’), probes and targets were labelled at 3’ end with Cy5 and Cy3 respectively:

- Probe P1: TTCACGATTCGCTATCTGA (19 mer)
- Probe P1T10: TTTTTTTTTTCACGATTCGCTATCTGA (27 mer)
- Poly A: AAAAAAAA (8 mer)
- Target: TTTCAGATAGCGAATCGTG

Relative Fluorescence Unit (RFU) measurement was obtained with an Axon Genepix 4000b Scanner with the scanner focus point set to 200 μm. Typically the scanner power was fixed at 100 and the photomultiplier tube (PMT) gain at 500 for crosslinking scan at 635 nm; for hybridization scan, the scanner power was typically set at 100 and PMT gain at 400. For comparison with chemical crosslinking, 535nm scan with scanner power set at 100 and PMT at 400 was used. We adjusted the scanner power and PMT gain when RFU signals were too weak or too strong. However, throughout this work, when comparisons were made, the same scan power and PMT gain were used to ensure the validity of comparison.

## Results and Discussion

GLAD-MACE substrate was consist of closely distanced nanowires with diameters ranging from 10 to 100 nm. Nanowires were vertically standing with height around 10 μm. We have shown previously that such nanowires are porous on the sidewalls [2]; and due to the high aspect ratio as well as porosity, the substrates to be ideal platform to increase surface loading capacity of biomolecules up to 250 fold [1]. However, such substrate faces severe problem of wicking. The substrate was spotted with 500 nL of Cy3 solution with liquid dispensed with a spotter, and the liquid spread into a spot with an average diameter of 9650 μm. On a similarly functionalized hydrophilic glass slide without nano features, the liquid only spread into a spot with an average diameter of 980 μm. On a functionalized hydrophobic surface, the droplet spreading was less than 200 μm. The severe liquid spreading limits the feature packing density of the substrate. More details on the morphology of the nanowires and the wicking characteristics on GLAD-MACE platform can be found in S2 File. This illustrates a severe problem of producing small functional spot on wicking platform such as GLAD-MACE with microarray printing technique.

To estimate the spreading of liquid, consider a simple case where wetting liquid would spread evenly on the surface and fill up to certain height of nanowires. A simple estimation of dispensing volume showed at most 50 pL can be dispensed to achieve a 100 μm diameter spot. However, this dispensing precision is difficult to achieve with average microarray printers. For example, BioRad BioOdyssey MCP system dispense volume ranging from 160 nL to 836 nL [25], while Arrayit microarray printer can deliver liquid down to 500 pL [26]. Among the manufacturers, Scienion’s top of line piezo nozzle is able to dispense liquid down to 50 pL [27]. A feasibility study by Scienion using piezo printing showed that the smallest diameter obtainable was 200 μm on GLAD-MACE substrate (Refer to S3 File for detailed experimental procedure). While the dispenser costs around USD 100K to 200K of purchasing such dispenser, our whole set up costs less than US$1K. For this reason, photo-attachment on wicking surface provides a practical and attractive solution to miniaturization.

It is generally agreed that biomolecules should not be subjected to harsh chemicals or photons of very short wavelengths to avoid damage to the molecules [28]. Among many crosslinkers, psoralen and diazirine have been used in many liquid phase biological systems for the investigation of DNA structures [29], DNA-protein [30] or protein-protein interactions [31], suggesting these chemicals possess good biocompatibility. In addition, both chemicals can interact with biomolecules in the UVA to UVB range, and not the high energy UVC range. Thus these two chemicals were chosen for the photo-attachment study.

It has been suggested that psoralen mainly crosslink to double strand DNAs through intercalating as it can insert its triple ring structure into the double strands DNA to form stable electronic orbital with the bases of nucleotides [32]. Among the nucleotides, psoralen may preferentially react with thymidine since it is able to couple more of its ring structure with thymine [32]. There are conflicting reports of whether psoralen can crosslink single strand DNA on solid substrate as in some cases crosslinking was not observed [14]. Diazirine has been used to crosslink protein with protein. Upon UV illumination, diazirine forms reactive carbene group which actively seeks N-H or C-H bond for insertion [33]. Diazirine can also react with aromatic compounds, such as bases in oligo DNA, when electron releasing group is present on the aromatic rings [34]. Unlike psoralen, diazirine has not been reported to crosslink with thymine favourably since there is no electrophilic groups on the aromatic ring, suggesting diazirine has less reactivity toward this nucleotide [34].

**Fig 1:**
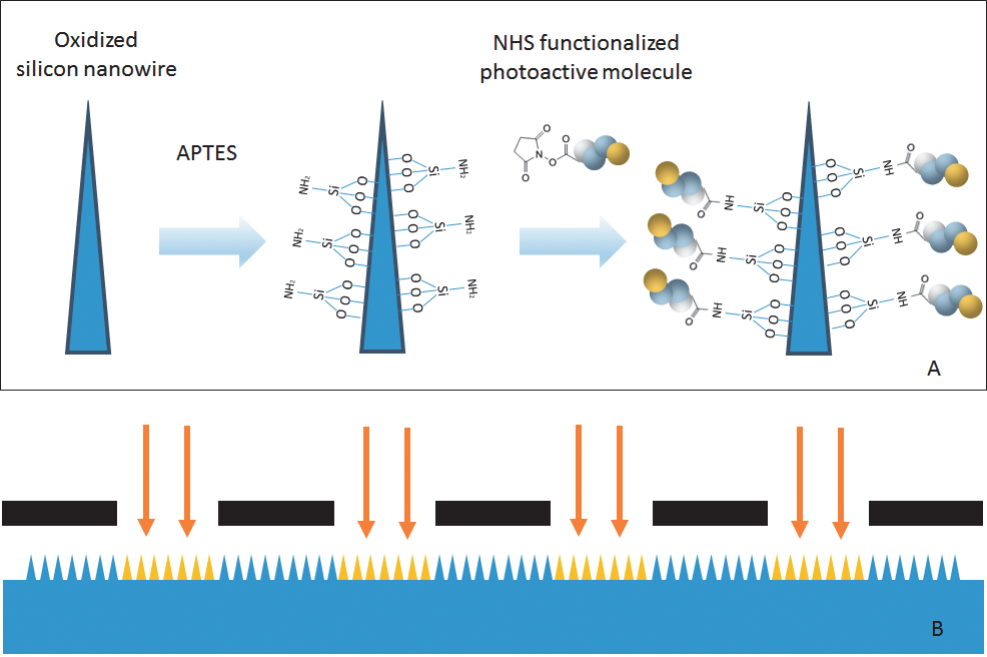
Experimental approach and and set-up for photo-attachment. A: Chemical reaction approach for photo-attachment of biomolecules on nanowire surface. B: Simple set up for photo-attachment. The black parts in Fig. 1B represents opaque region of photomask. The yellow region represents photo-attached nanowires.

Two approaches of photo-attachment can be employed to attach biomolecules onto nanowire surfaces. The first approach is to conjugate photo-attachable molecules on the biomolecule itself through a secondary functional group, such as NHS; and the conjugated biomolecules will crosslink with surface under illumination. However, this requires some biomolecules to be chemically modified to present a reactive group which may be costly [20]. More importantly, prolonged reaction time as well as subsequent purification are often required, which may increase the risks of damaging fragile biomolecules. In view of these considerations, we chose to conjugate photo-attachable molecules on nanowires, Fig. 1A shows schematically the conjugation approach we used to link photo-attachable moieties on nanowires. Fig. 1B illustrates the set up used for photo-attachment.

**Fig 2:**
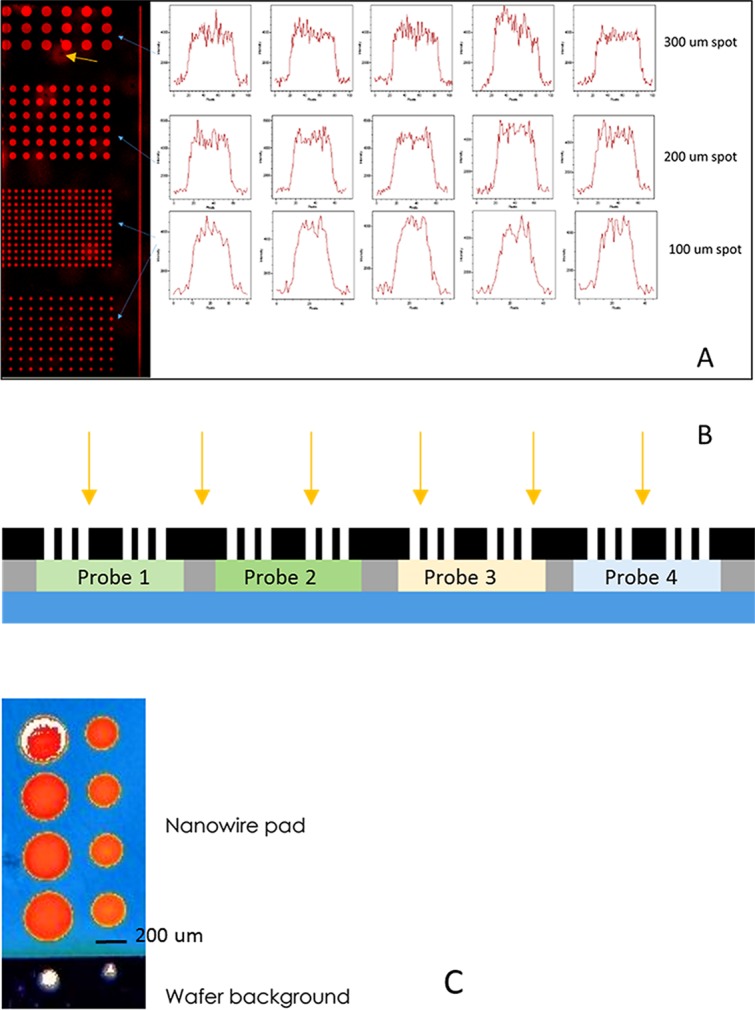
Results of photo-attached spots of different diameters. A: Array Fabrication of photo-attached spots of various diameter, yellow arrow pointing anomaly surface area resulted from nanowire fabrication process. B: Schematic diagram showing the approach to immobilize different probes at different positions separated by specially designed gaskets (black portion) and C: Piezo dispensing by Scienion AG, with permission to reproduce image.

Fig. 2 shows the results of our photo-attachment experiments and it shows that our method can produce miniaturized detection sites down to 100 μm with good fidelity (lateral spread < 20%). Note that with photo-attachment, the spot size is controlled by the diffraction [35] and reflection of the light source. The Si nanowires of the GLAD-MACE platform are highly absorbance to light (“black Si”) [36] and this will maximize the absorption of light for photo-attachment of analytes, and minimize the reflection of light outside the illuminated spot and hence ensure good spot size control. This degree of fidelity is similar to that obtained by arrayer on flat hydrophobic substrate [26], and better than printing on hydrophilic substrate [3,37]. No doughnut profile or crystallization induced uneven immobilization was observed. Note that even though a further miniaturization of the detection sites is possible with our method, we have decided to fix the spot size at 100 μm as the pixel size of typical microarray scanner is 5 μm and detection sites less than 100 μm may not produce enough pixels to make a statistically reliable readout.

To immobilize different probes at different locations of a GLAD-MACE platform, two approaches can be used. The first approach is to immobilize one kind of probe at one exposure step. By shifting masks between exposures, different probes can be immobilized at various locations. This approach is particularly suitable for photo-attachment with chemicals that can react in shorter time scale. Another approach would be to segregate probes to different portions on the surface, and expose the substrate at one go. The segregation can be achieved by attaching gaskets on the substrate with PDMS, this approach is illustrated schematically in Fig. 2B. Note that a feasibility study by Scienion using piezo printing showed that the smallest diameter obtainable was 200 μm on GLAD-MACE substrate, as shown in Fig. 2C.

**Fig 3:**
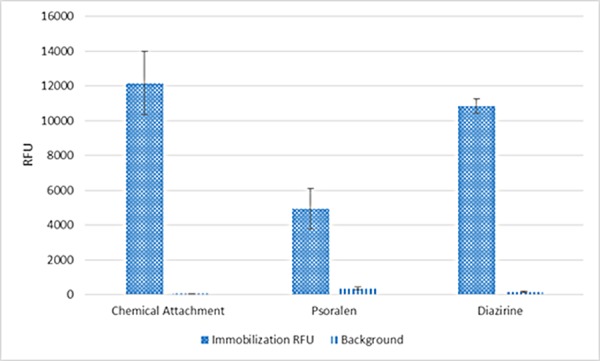
Comparison of crosslinking efficiency between chemical immobilization and photo-attachment.

As we have demonstrated the successful implementation of photo-attachment of analytes to the GLAD-MACE platform, we next present results of a comprehensive study on the influence of the various factors on the photo-attachment process. We first compare the efficiency of photo-attachment to that of chemical crosslink. Chemical attachment was performed with EDC to crosslink carboxyl group and amine group, which is a fast and highly efficient reaction. As shown in Fig. 3, photo-attachment with diazirine produced roughly similar extent of crosslinking as chemical attachment, indicating diazirine reacts efficiently with oligos on the solid interface. The amount of attached molecules on diazirine functionalized surface can be further increased by increasing the exposure time. For psoralen, the photo-attachment efficiency is generally lower, probably due to single strand instead of double strands were used.

**Fig 4:**
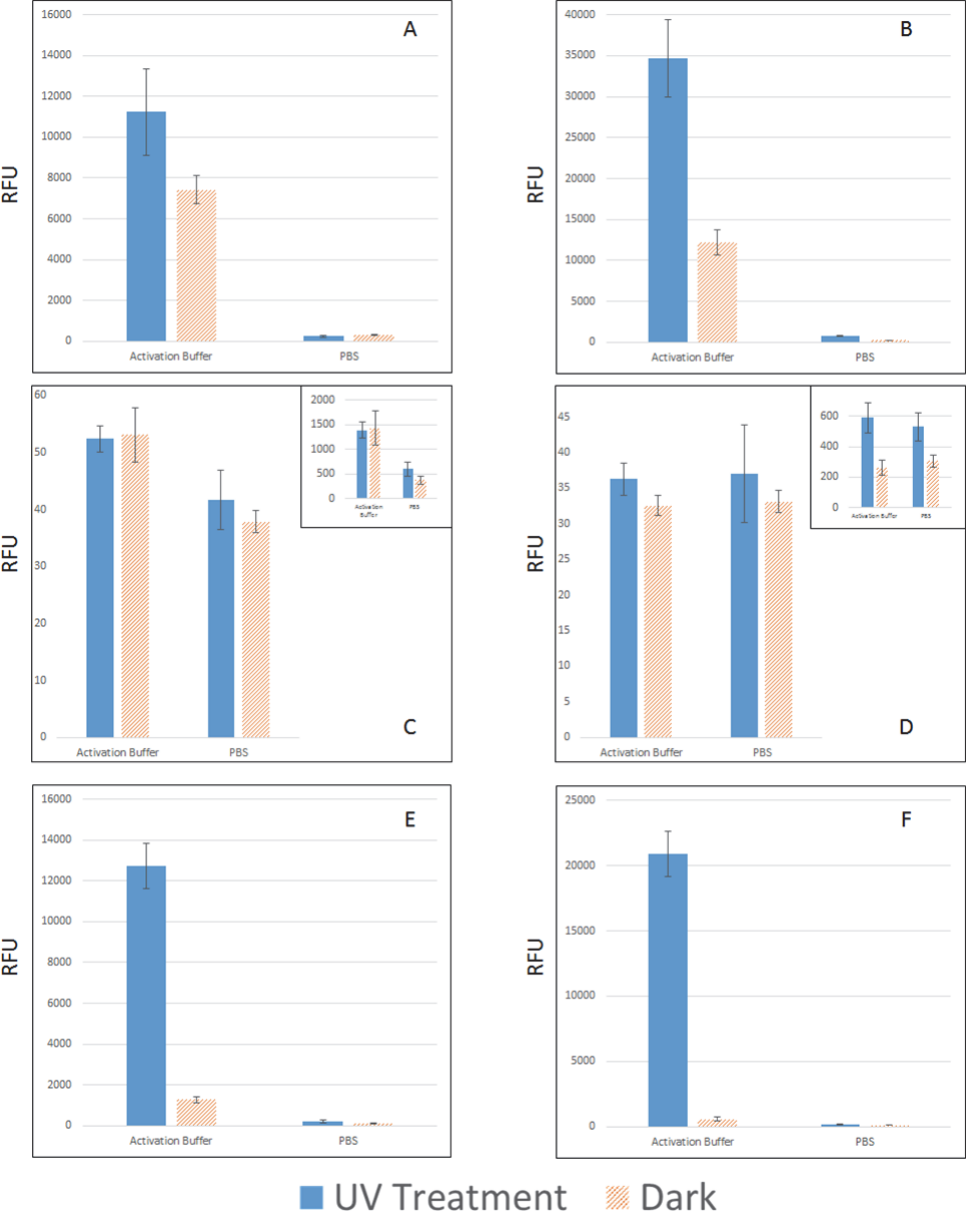
Photo-attachment to crosslink sense oligo on nanowire substrates. A: Psoralen functionalized nanowire substrate, B: Diazirine functionalized nanowire substrate, C: Psoralen functionalized polished silicon substrate with the inset shows the same chip scanned at maximum power and PMT gain, D: Diazirine functionalized polished silicon substrate with the inset shows the same chip scanned at maximum power and PMT gain, E: Psoralen functionalized with passivated substrate, and F: Diazirine functionalized with passivated substrate.

We also carried out photo-attachment experiments in two different buffers. Fig. 4A and B show that, in PBS buffer (one of the commonly used buffers for microarray printing), no crosslinking was observed by photon radiation on psoralen functionalized nanowires; and only slight crosslinking on the diazirine functionalized nanowires was observed. However, when activation buffer was used, a significant increase of RFU was observed in both illuminated and unilluminated substrates (see Fig. 4A and B, Activation Buffer). A similar buffer effect has been observed by Ahn et al. [38] when psoralen was functionalized on dextran, where a lower pH helped photo immobilization of oligo on dextran.

With activation buffer, although the RFU signal on UV treated substrates increased dramatically, there is also significant RFU increase from unilluminated samples, which represents undesired background. Such background was extremely difficult to eliminate through washing. For instance, 10% SDS heated up to 60°C was used to wash for up to 4 hours only reduced the background RFU marginally. As wicking substrate produces large area of interaction, the undesired attachment of DNA may give rise to higher chances for the biomolecules to interact with unintended surface. This problem was prevented by passivating the surface with adipic acid after psoralen and diazirine have been crosslinked, as shown in the substantial reduction in the background signal in Fig. 4E and F. The signal to background ratio is more than 10 folds for psoralen and more than 20 folds for diazirine.

The same photo-attachment procedure was also performed on non-wicking oxidized silicon substrates similarly functionalized with psoralen and diazirine with both activation buffer and PBS buffer at a concentration of 20 μM. When scanned at the same power and MT gain, they showed negligible photo-attachment as shown in Fig. 4C and D. A rougher wicking surface reduces the reflection of illumination and this resulted in the much higher photo-attachment on the GLAD-MACE surface as compared to the polished silicon substrate [39,40]. Another reason is the nanowire wicking substrate has a much larger amount of surface bound amine group for photo-attachable moiety grafting. We have measured the surface bound amine with the consumption of NHS-Dylight 550 and the results are shown in Table 1. For a unit substrate size, the nanowire substrates consumed about 40 times more NHS-Dylight 550 compared to flat silicon. This result strongly suggest photo-attachment gain additional advantages on wicking substrates.

**Table 1:** Estimation of surface bound amine group for flat Si wafer and GLAF-MACE (Chip1 and Chip 2) surfaces.

	Substrate Size (cm^2^)	Reacted Dye Amount (nmol)	Unit Area Amine Ratio by Dye Consumption
Flat Wafer	20	3.65	1
GLAD-MACE Substrate 1	0.25	2	43.8
GLAD-MACE Substrate 2	0.25	1.85	40.7

As wicking enhances surface for reaction with biomolecules, it is vital to reduce non-specific surface adsorption on such surface. Here we investigate the reason for photo-attachment to happen in activation buffer but not PBS, as well as the explanation of why adipic acid can reduce non-specific adsorption. Photo-attachment can be viewed as a two-step process: firstly biomolecules need to come to the proximity of nanowires grafted with photo-attachable moieties, and then the photo-attachment reaction will link those molecules on the nanowires. A low pH environment may be conducive to photo-attachment reaction as there are reports of oligonucleotide went through structure change in acidic environment [41]. But that requires specific sequence designs in oligonucleotides which are not present in our case. We suspected the effects of activation buffer and adipic acid passivation shown in Fig. 4 may be related to the first step.

**Fig 5:**
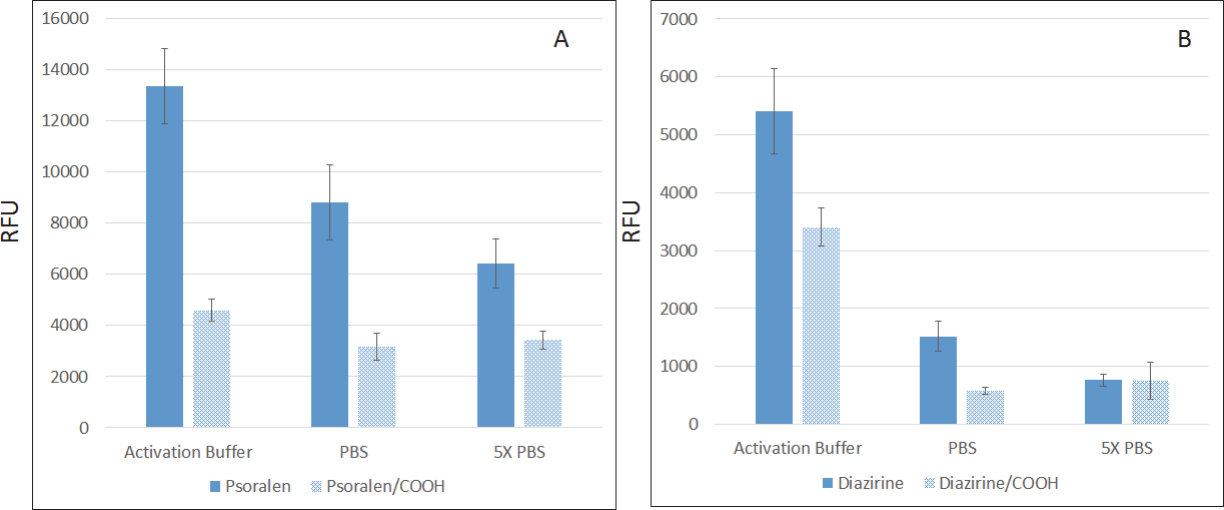
Results on nonspecific adhesion study. A: Psoralen functionalized nanowire surface and B: Diazirine functionalized nanowire surface.

An adhesion study was performed with 20 μM probe sequence incubated for 3 hours on GLAD-MACE platform and then lightly washed. The sample was subsequently scanned at PMT = 700 and Power = 100. As shown in Fig. 5A and B, for both psoralen and diazirine functionalized substrates, adsorption is strongly enhanced in activation buffer compared to PBS, which could explain the apparent enhanced performance of photo-attachment in activation buffer (Solid Bars). In addition, the adsorption in 5X PBS is weakest. As the ionic strength is stronger in the 5X PBS buffer as compared to PBS buffer, this implies that adsorption is promoted by electrostatic interaction. In 5X PBS buffer, the electrostatic interactions were reduced and this resulted in the reduction of adsorption. With activation buffer at low pH, adsorption was much enhanced. This effect has been observed with amine functionalized surfaces previously [42], where amine groups were protonated at low pH. In our case, we suggest that both Si-OH groups and remaining amine groups can be protonated to interact with the negatively charged DNA backbones. When adipic acid was grafted, carboxyl group will be presented on the surface, and a general reduction of adsorption was observed. The reduction effect is much stronger in PBS compared to 5X PBS, which also implies the reduction is due to electrostatic interaction as surface bound carboxyl group will produce negative charge which can repel negatively charged DNA backbones.

**Fig 6:**
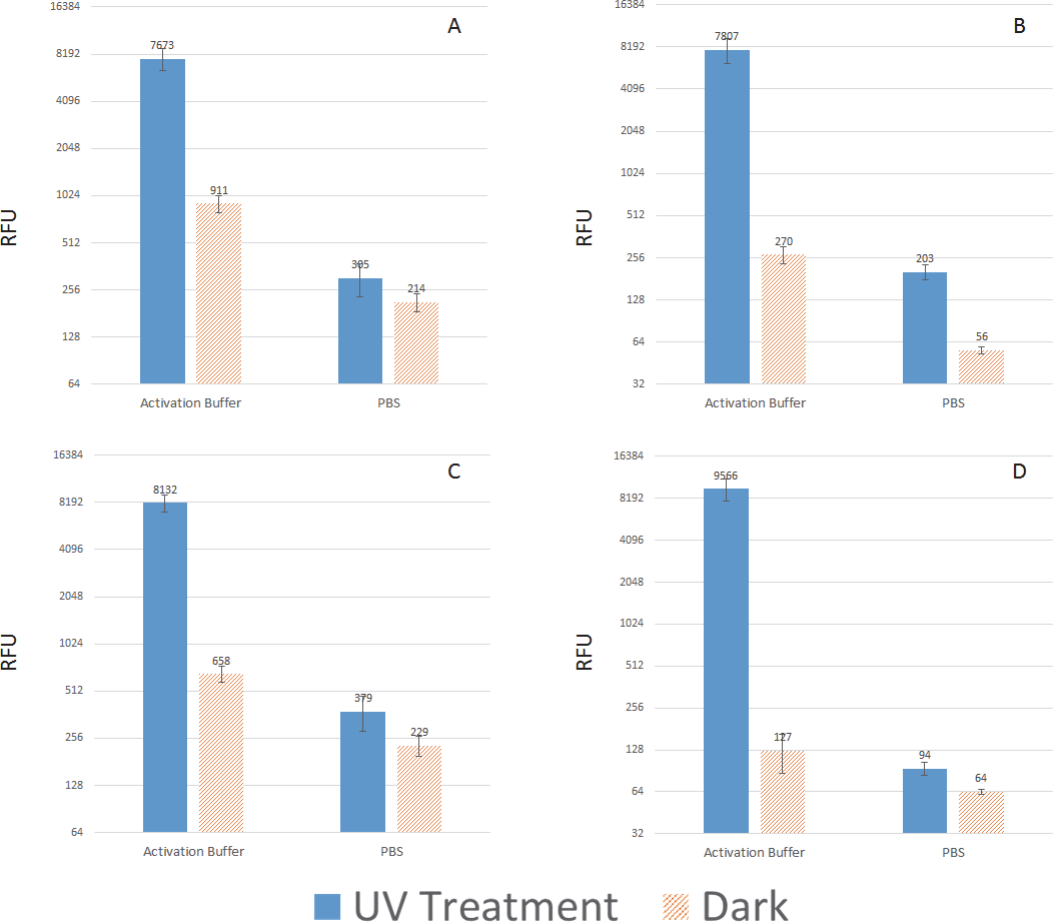
Hybridization of target oligo on photo-attached substrates. A: Psoralen functionalized substrates, B: Diazirine functionalized substrates, C: Psoralen functionalized substrate with passivation, and D: Diazirine functionalized substrate with passivation.

Hybridization was tested with photo-attached substrates to demonstrate the structural integrity and bioactivity of crosslinked oligonucleotides. A 1 μM of target sequence was used for hybridization. Fig. 6A and B shows the hybridization results on psoralen and diazirine functionalized nanowires, and it is clear that the structure and function of crosslinked probes remain intact. Furthermore, Fig. 6C and D show hybridization on adipic acid passivated psoralen and diazirine substrates, hybridization background dropped 1.5 to 2 folds, which may help improving the limit of detection for such substrates.

It is important to examine that for any substrate operates with immobilization method must produce a platform that has discrimination power to distinguish similar nucleotides. Solid substrate has always faced challenges to distinguish similar nucleotide sequences mainly due to the interaction of probe and target molecules with solid interface. As such, discrimination between targets with single base mismatch has become a well-accepted standard to examine discrimination power. It must be noted, even when immobilization method and solid substrate are chosen, the discrimination can still vary largely depending on the design of probe [43], the hybridization environment, the detection method [44] as well as the location of the single nucleotide mismatch.

**Fig 7:**
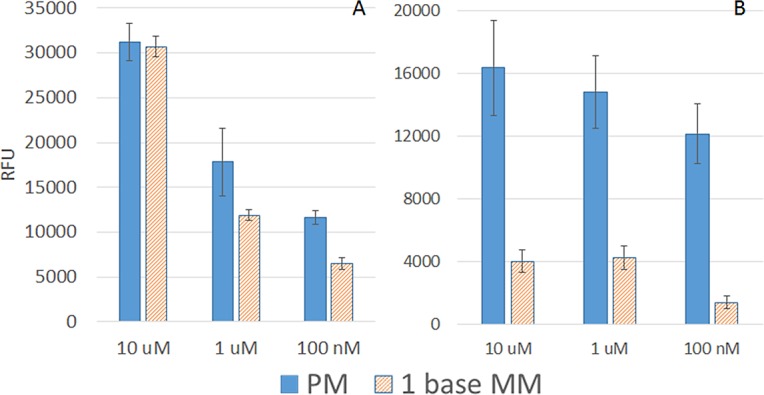
Results of discrimination of nucleotide with single base mismatch. A: Without formamide and B: With formamide on Psoralen functionalized surface.

We have carried out the discrimination experiments and compare it with reports in the literatures. We used exactly the same probe sequence except the poly G region as in reference [45] and the same target sequences, and the results are shown in Fig. 7.

- Probe: TTT TTT AAC TAT ACA ACC TAC TAC CTC A
- Perfect Match target (PM) Cy3 labelled miRNAhsa-let-7a: 5’ UGA GGU AGU AGG UUG UAU AGU U
- 1 base pair Mismatch (MM) Cy3 labelled miRNAhsa-let-7f: 5’ UGA GGU AGU AGA UUG UAU AGU U

We have compared discrimination power at 3 concentrations, 10 μM, 1μM and 100 nM of PM target and MM target. It is noted the discrimination power increases with decreasing target concentration, which is likely due to the reduced hybridization driving force (chemical potential). On the psoralen surface, without formamide (Fig. 7A), we noted a discrimination around 2 fold at 100 nM. Our results are similar to previous report [45], where the single base pair MM has a discrimination ratio around 1.5 fold at 100 nM. According to Fuchs et al. [46], the discrimination measured with SNP oligonucleotides at 37°C without formamide was around 0.9 on biochip surface. According to Oh et al. [47], the discrimination ratio for various surfaces for internal mismatch ranges from 0.2 to 0.6, we find our results in line with literatures. To further improve on SNP discrimination power, we added 20% formamide while hybridization (see Fig. 7B). It is noted that the discrimination ratio becomes very high around 100 nM (around 10 fold). We have repeated the discrimination without formamide on diazirine functionalized surface and the results are similar to that on psoralen functionalized surface. These results demonstrate our substrate with photo attachment strategy provided a platform with good specificity.

Psoralen and diazirine interacts with oligonucleotides differently. Psoralen has been known to intercalate with DNA strands and thus favours crosslinking with double strand DNA. In addition, it is known that thymine is the preferred interaction site of psoralen. On the other hand, diazirine crosslinks through a less selective approach. It forms carbene reactive group and inserts itself into C-H or N-H bond upon illumination. To explore the characteristics of the chemicals, we have synthesized 3 probes, the most basic one, labelled P1, and the second type of probe with 8 additional thymines at 5’ end, labelled as P1T10. The third kind of probe is a half hybridized probe between a poly A (5’AAAAAAAA 3’) with P1T10 at 4°C, labelled as P1T10A8.

**Fig 8:**
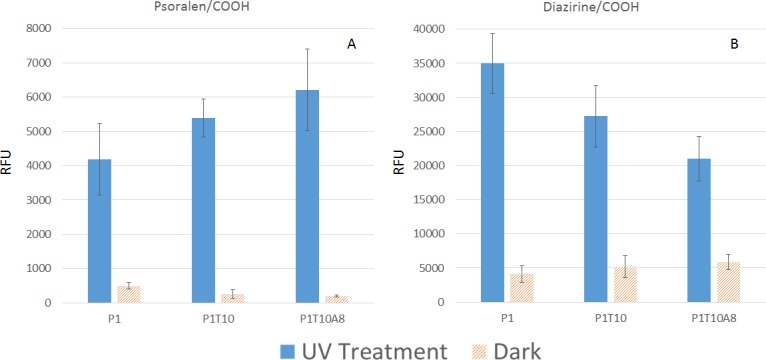
Probe crosslinking on nanowires with different probe structures. A: On psoralen functionalized surface passivated with adipic acid and B: On diazirine functionalized surface passivated with adipic acid.

Fig. 8A and B show contrasting behaviours of crosslinking efficiency can be increased on psoralen functionalized surfaces, with P1T10A8 generating the highest RFU. On diazirine functionalized substrates, the reverse trend can be seen. When probes were varied from P1 to P1T10A8, two significant changes occurred. Firstly, the number of thymines increased with poly Ts appended on the tail, and a half hybridized structure will form between P1T10 and poly A. Secondly, the size of the probe will increase dramatically, with radius of gyration almost linearly related to number of nucleotides under the same condition [48]. For psoralen functionalized surfaces, the two effects compete with each other, and the RFU increase can be explained by the preferential reactivity of psoralen to thymine as well as to double strand DNA. For diazirine functionalized surfaces, since there was no preferential reactivity, the increased size of probes P1T10 and P1T10A8 poses more steric hindrance for crosslinking, as larger molecules take up larger footprints on the surface. The results indicate that probe design should be taken into consideration. For chemicals like psoralen, to improve crosslinking efficiency, probes with hairpin structures may be employed in the future. However, diazirine should be the chemical of choice if multiple probes with different sequence needs to be immobilized at equal efficiency. It is also advisable to keep probes at similar length so that the size effect will influence different probes similarly.

**Fig 9:**
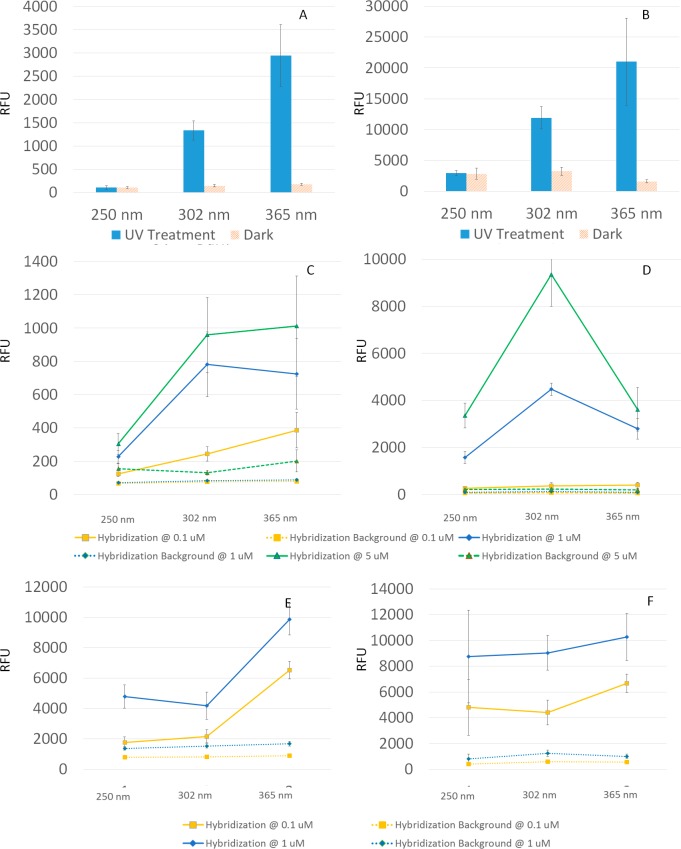
Photo-attachment results on different surfaces. A: On psoralen/COOH surface and B: On diazirine/COOH surface at wavelengths of 254 nm, 302 nm and 365nm with a probe concentration of 20 μM. C and D: Hybridization on nanowires crosslinked with 20 μM probes, E and F: Hybridization on nanowires crosslinked with 2 μM probes, Note that the bars indicate sense crosslinking and the lines indicate hybridization.

It is reported that regular micro or nanostructures can wick [49] and can absorb UV light at certain wavelength [50]. For such surfaces, choosing the wavelength represent another level of control for photo-attachment process. Therefore, it is worth investigating if photo-attachment is achievable through a range of wavelengths for the GLAD-MACE platform. Psoralen and diazirine have been reported to crosslink biomolecules in solution phase most favourably at wavelength around 360 nm [33]. In this work, three wavelengths (254nm, 302nm and 365nm) were employed to introduce photo-attachment. Fig. 9A and B show that for both psoralen and diazirine surfaces, crosslinking signals increased as wavelength shifted to longer wavelength, indicating the amount of crosslinked probes increased at longer wavelength of illumination. This is in good agreement with the reported results obtained in from solution phase [30,31].

Hybridization experiments were carried out subsequently at concentration of 1 μM. At 1 μM the hybridization signal only increases as the illumination wavelength increase from 254 nm to 302 nm. With further increase in the wavelength to 365 nm, the hybridization signal for the psoralen functionalized surface levelled off, and for the diazirine functionalized surfaces, a reduction of the hybridization signal can be observed. This phenomenon could be caused by two possible scenarios: either not enough target was present at the time of hybridization or the surface has been over crosslinked at 365 nm thus surface hindrance effect occurred.

To test the first hypothesis, we have increased target concentration of 5 μM with a longer hybridization time of 3 hours and found the same trend was repeated (Fig. 9C and D, green lines). However, at lower target concentration of 0.1 μM, continued increase in the hybridization signal can be observed for both kinds of surfaces (Fig. 9C and D, yellow lines), which implies a lower target concentration does not crowd the surface. To further test our conclusion, we performed photo-attachment at a lower probe concentration of 2 μM (Fig. 9E and F) and hybridization at target concentrations of 0.1 μM and 1 μM. Under this condition, both target concentrations generated an increasing hybridization signal with longer wavelength, which means reducing probe concentration could effectively reduce hindrance effect.

There are reports indicating that hybridization efficiency decreased when surface probe density exceeded certain threshold [51–54]. It is also shown that to achieve stable hybridization, DNA molecules needs to be in a space of 2–6 times of molecular size [55]. Our results show that careful optimization must be performed when photo-attachment is used to crosslink biomolecules such that potential surface hindrance effect can be avoided.

## Conclusion

The results presented in this study demonstrate photo-attachment of biomolecules on GLAD-MACE platform with low energy UV source. We used simple lithography setup and commercially available chemicals to ensure the platform can be produced and used cost effectively. Various factors that affect the photo-attachment were studied. Our study showed that photo-attachment can produce miniaturized detection sites on wicking surface, enabling hydrophilic high aspect ratio nanostructured surface to be used for with miniaturized detection sites. The technique help to avoid high set up cost of the state of art microarray printing apparatus. Note that even though we have only demonstrated photo-attachment on GLAD-MACE platform, we expect photo-attachment can be employed on other wicking surfaces.

## Supporting Information

S1 File Experimental Data for time course of crosslinking and hybridization of psoralen and diazirine functionalized substrates. Figure A, Results of time course of crosslinking and hybridization. A: On psoralen functionalized substrate and B: On diazirine functionalized substrate.
(DOCX)

S2 File Wicking properties of GLAD-MACE Nanowires. Figure B, Wetting properties of nanowire substrate. A: Top view of nanowire substrate, B: Side view of nanowire substrate, scale bars are of 20 μm, and C: Wicking behaviour of aqueous solution on nanowire substrate. The trend line shows data fit for hemiwicking diameter with respect to t^0.25^
(DOCX)

S3 File Experimental Conditions for Piezo Nozzle Printer. (DOCX)
