# Supplementary material for: Photo-Attachment of Biomolecules for Miniaturization on Wicking Si-Nanowire Platform
Source: PLoS One. 2015 Feb 17;10(2):e0116539. doi: 10.1371/journal.pone.0116539 (PMC4331555; doi:10.1371/journal.pone.0116539)
Supplement: S1 File — Figure A, Results of time course of crosslinking and hybridization. A: On psoralen functionalized substrate and B: On diazirine functionalized substrate. (DOCX) [file pone.0116539.s001.docx]

**S1 File (Experimental Data for time course of crosslinking and hybridization of psoralen and diazirine functionalized substrates)**

Figure A shows the crosslinking time course for psoralen (A) and diazirine (B) functionalized surfaces. The hybridization for the time course was performed at a concentration of 0.1 μM. For psoralen functionalized surfaces, the RFU signals increased until it reaches a plateau around 40 minutes. For diazirine functionalized surfaces, the signal continues to rise even after 60 minutes, which suggests diazirine may have higher reactivity for photo-activation.


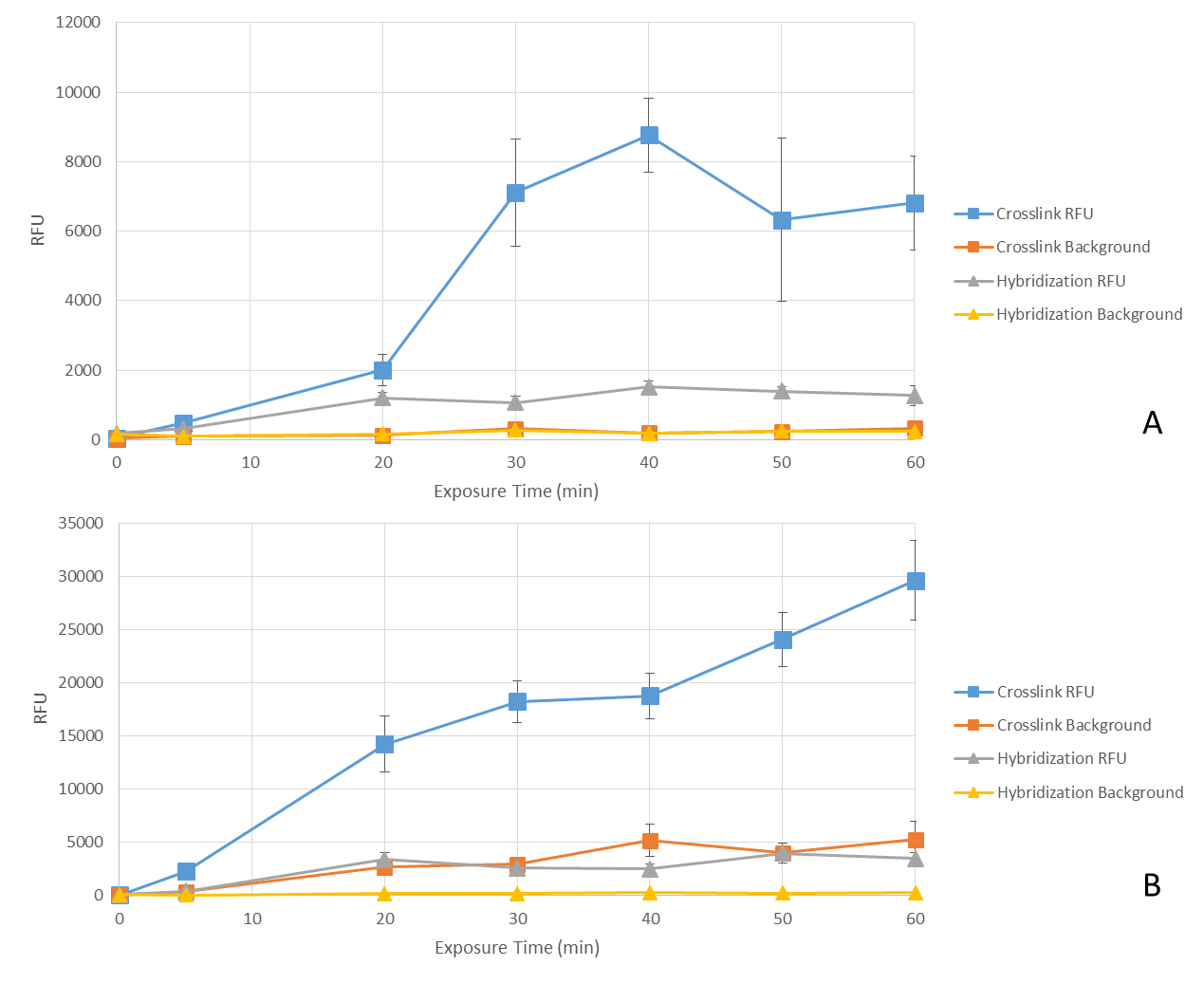


Figure A. Time course of crosslinking and hybridization of psoralen functionalized substrate (A) and diazirine functionalized substrate (B).
