# Supplementary material for: Photo-Attachment of Biomolecules for Miniaturization on Wicking Si-Nanowire Platform
Source: PLoS One. 2015 Feb 17;10(2):e0116539. doi: 10.1371/journal.pone.0116539 (PMC4331555; doi:10.1371/journal.pone.0116539)
Supplement: S2 File — Figure B, Wetting properties of nanowire substrate. A: Top view of nanowire substrate, B: Side view of nanowire substrate, scale bars are of 20 μm, and C: Wicking behaviour of aqueous solution on nanowire substrate. The trend line shows data fit for hemiwicking diameter with respect to t0.25 (DOCX) [file pone.0116539.s002.docx]

**S2 File. (Wicking properties of GLAD-MACE Nanowires)**

The Si nanowire based substrate fabricated with the GLAD-MACE technique are shown in Fig. B, A and B. The nanowires usually have a length of more than 10 μm, with a diameter ranging from 10 nm to 100 nm. The nanowires have a solid cores and mesoporous layer on the surface. Upon drying, nanowires may bunch together to form clusters. The spreading of the liquid was measured with respect to time and shown in Fig. B, C. The droplet spreads rapidly in the initial 1^st^ second and then the droplet size reaches a plateau. We have observed the film continued to extend for more than 20 seconds. Our experiment results agreed very well with that reported by Kim *et al*. (“Experimental study of drop spreading on textured superhydrophilic surfaces”, *Physics of Fluids*, 2013, **25**, 9) and their model on structured wicking surface.


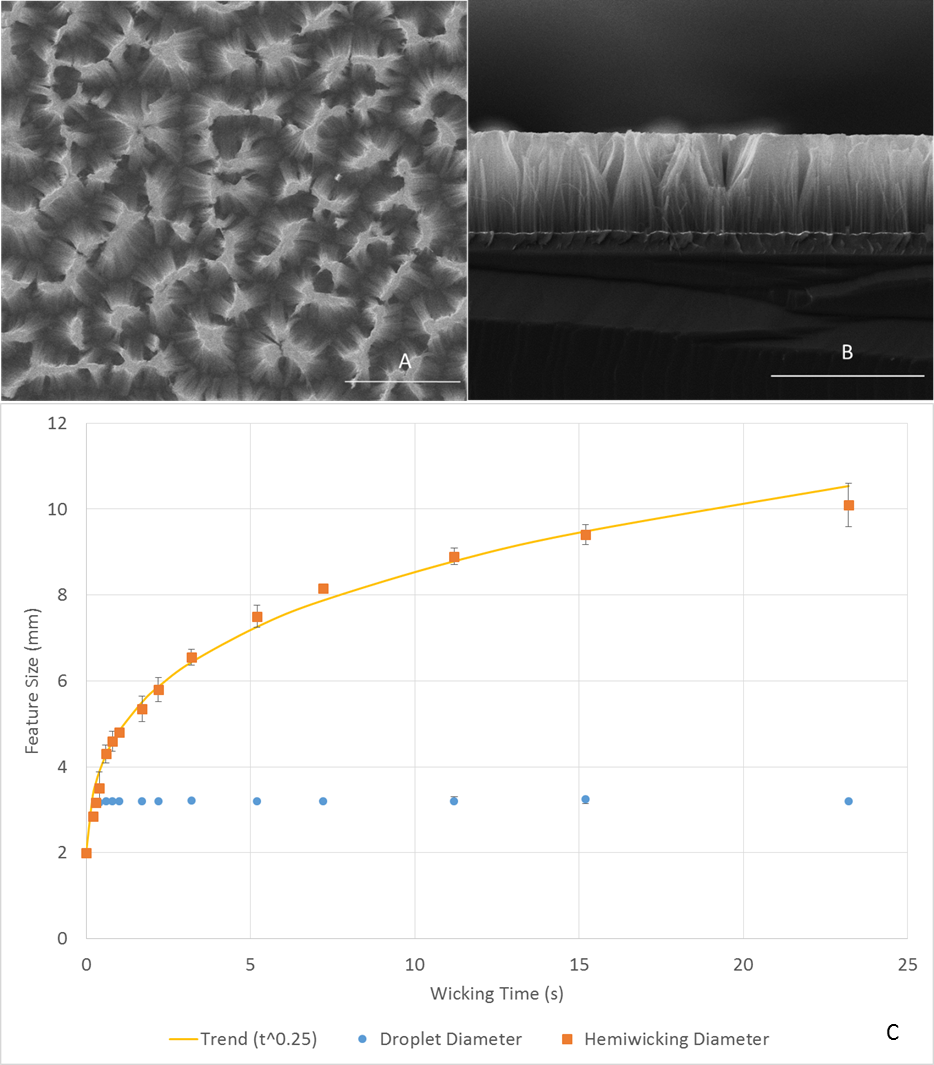


Figure B. Top view of nanowire substrate (A), side view of nanowire substrate (B), scale bars are of 20 μm, and (C) wicking behaviour of aqueous solution on nanowire substrate. The trend line shows data fit for hemiwicking diameter with respect to t^0.25^
