# Supplementary material for: Photo-Attachment of Biomolecules for Miniaturization on Wicking Si-Nanowire Platform
Source: PLoS One. 2015 Feb 17;10(2):e0116539. doi: 10.1371/journal.pone.0116539 (PMC4331555; doi:10.1371/journal.pone.0116539)
Supplement: S3 File — (DOCX) [file pone.0116539.s003.docx]

**S3 File (Experimental Conditions for Piezo Nozzle Printer)**

In order to shrink the spot size, we have contacted Scienion AG, a leading manufacturer for high precision robotic piezo nozzle printer that can dispense smallest droplet in the market (50 picoliter volume accurately) to work on our substrates. The smallest dimension Scienion AG can achieve was 200 μm, while on non-wicking Si substrate, the spot are much smaller. Therefore, to further reduce the spot size, it is clear that we have to rely on other approaches rather than the dispensing method.

Experiment Condition:

Nozzle PDC 50; Coating type 3, (#8862; 99V, 9 µs), Drop volume: 55pL

Humidity: 75 %, Temperature: 24 °C and Source Plate Temperature: 10,5 °C

The liquid was incubated on GLAD-MACE substrate for 2 hours, and scanned directly without washing. However, Scienion was not able to confirm whether the droplet has been dried during this time. If so, the immobilization efficiency for oligonucleotide immobilization with droplet dispensing would be impaired.

Note that the 55 pL is the smallest volume can be dispensed, Scienion pointed out under this extreme condition, only liquid with surface tension similar to water can be dispensed, which would limit the applicable range of reagent could be used.
